# Status Epilepsy Syndromes Made Easy: Pediatric Perspectives

**DOI:** 10.3390/children12121709

**Published:** 2025-12-17

**Authors:** Kam Lun Ellis Hon, Alexander K. C. Leung, Karen K. Y. Leung, Alcy R. Torres

**Affiliations:** 1 Department of Paediatrics, CUHK Medical Centre, The Chinese University of Hong Kong, Shatin, Hong Kong; 2 The Paediatric Intensive Care Unit (PICU), Hong Kong Children’s Hospital, Kowloon, Hong Kong; 3 Department of Pediatrics, Alberta Children’s Hospital, University of Calgary, Calgary, AB T2N 1N4, Canada; aleung@ucalgary.ca; 4 Division of Pediatric Neurology, Boston Medical Center, Boston University Chobanian and Avedisian School of Medicine, Boston, MA 02118, USA; artorres@bu.edu

**Keywords:** FIRES, NORSE, new onset refractory status epilepticus, SE, RSE

## Abstract

**Introduction**: Refractory Status Epilepsy Syndrome is a heterogeneous group of diseases associated with status epilepsy. Literature and definition have been conflicting and confusing in terms of their nomenclatures. New-onset refractory status epilepticus (NORSE) is a syndrome characterized by new onset refractory seizures in a previously health child. Febrile infection-related epilepsy syndrome (FIRES) is a similar syndrome now considered a variant of NORSE and is defined as a febrile event taking place between twenty-four hours and two weeks prior to the commencement of refractory status epilepticus. An autoimmune or inflammatory etiology is often implied in both conditions because infection is rarely identified. **Aim**: This review provides an update on hypotheses, etiology, pathophysiology, clinical features, diagnosis, laboratory evaluation, treatment, and perspectives for NORSE/FIRES. **Methods**: A PubMed Clinical Queries search is performed using keywords of NORSE and FIRES, on human subjects up to May 2025. All reviews, systematic reviews, case series and case reports were included. **Results**: Seizures are typically recalcitrant in NORSE/FIRES. Treatments include anti-seizure medications (ASM), ketogenic diet, immunotherapy (intravenous immunoglobulin ± plasmapheresis ± corticosteroid). The prognosis is usually poor. Most children would suffer refractory epilepsy and associated cognitive impairment if they survived. Guidelines and new consensus on NORSE/FIRES terminology have aided clinicians in managing status epilepticus in a previously healthy child that occurs ± a minor febrile episode. When an autoimmune or paraneoplastic condition is subsequently identified, the condition will be named accordingly. **Conclusions**: NORSE and FIRES are similar conditions except that vagus nerve stimulation appears to be more efficacious in NORSE than FIRES. We propose to define these heterogeneous and confusing conditions as “**NOSES**” as a two-criteria syndrome: **N**ew **O**nset + **S**tatus **E**pilepticus **S**yndrome, lasting for over 24 h despite the use of two standard ASM. Autoimmune, paraneoplastic and infectious encephalitis are specific diagnoses of NOSES with etiology subsequently identified.

## 1. Introduction

Status epilepticus (SE) is a neurological emergency characterized by recurrent or prolonged seizures without full recovery of consciousness between episodes. The International League Against Epilepsy (ILAE) defines SE as a condition with prolonged seizures, lasting beyond five minutes for convulsive SE and ten minutes for non-convulsive SE [1,2]. New Onset Refractory Status Epilepsy (NORSE), as the name implies, refers to an epilepsy syndrome manifests in previously healthy patients in the absence of any history of epilepsy and/or neurological disorders [3]. SE syndromes are classified based on clinical features, EEG patterns, etiology, and response to treatment [1,2]. The primary categories include Convulsive Status Epilepticus (CSE), characterized by prolonged tonic–clonic seizures. It is the most common and severe form, often requiring immediate intervention. Non-Convulsive Status Epilepticus (NCSE) includes absence SE and complex partial SE, presenting with altered consciousness and subtle motor signs. Refractory Status Epilepticus (RSE) is SE that persists despite first-line benzodiazepines and second-line Anti-Seizure Medications (ASM). Super-Refractory Status Epilepticus (SRSE) is SE that continues or recurs 24 h after the onset of anesthetic therapy. Pediatricians and acute care physicians have to manage previously healthy children with new-onset refractory seizures from time to time. Literature has been conflicting and does not give clear directions as to its management [3]. NORSE shares similarities with other pediatric seizure syndromes, including Idiopathic Hemiconvulsion-Hemiplegia and Epilepsy Syndrome (IHHES) and Febrile Illness-Related Epilepsy Syndrome (FIRES) [3,4,5]. Febrile Infection-Related Epilepsy Syndrome (FIRES) is a rare, catastrophic SE syndrome of new-onset seizures in previously healthy children with a preceding febrile infection-like illness between twenty-four hours and two weeks before the onset of refractory seizures, regardless of fever at seizure and age of the patient [4,6]. Seizures begin and become status epilepticus in twenty-four hours to two weeks, which can be simple, complex, or secondary generalized. The etiology for NORSE/FIRES is unknown, and the syndrome may be caused by an autoimmune mechanism.

Some of the patients with NORSE/FIRES are subsequently diagnosed as having a specific encephalitis/encephalopathy syndrome, and may be reclassified under such categories as autoimmune, paraneoplastic, and infectious encephalitis or encephalopathy with status epilepticus (SE). Encephalitis literally means inflammation of the brain [7]. The incidence of acute encephalitis is about seven cases per 100,000 people per annum [8,9,10,11]. Symptoms of acute encephalitis include fever, headache, reduced or altered consciousness, neck stiffness, confusion, vomiting, seizures, hallucinations, disturbed speech, memory loss, and hearing deficits [12,13]. Infectious etiologies of acute encephalitis include viruses (e.g., rabies, herpes simplex virus, poliovirus, and measles), bacteria, fungal pathogens, or parasites (e.g., malaria, Lyme disease, *Bartonella henselae*, Mycoplasma) [14,15,16,17,18]. Non-infectious causes include acute demyelinating disseminated encephalitis (ADEM), autoimmune diseases, and cryptogenic [7]. Diagnosis is typically based on symptoms of fluctuating or altered level of consciousness and supported by blood tests, neuroimaging (e.g., brain scan, MRI), and analysis of cerebrospinal fluid (CSF) for at least 24 h [13,19,20]. EEG is important in monitoring brain activity, and CSF polymerase chain reaction (PCR) testing to detect viral DNA may facilitate diagnosis. PCR is not helpful for diagnosing bacterial, fungal, and parasitic encephalitis. Therapeutic management is often challenging and may include specific antiviral drugs (e.g., acyclovir), ASM, and corticosteroids [13]. Intravenous immunoglobulin is often used for childhood encephalitis although its effectiveness is unclear [7,13]. No firm conclusions could be drawn because of insufficient clinical evidence [7,21].

Non-infectious encephalitis includes paraneoplastic, autoantibody, and miscellaneous [7]. Limbic encephalitis is confined to the limbic system of the brain and is confirmed by magnetic resonance imaging (MRI) abnormalities in the medial temporal lobes. Symptoms include disinhibition, disorientation, memory loss, and convulsions. Early immunotherapy (glucocorticoids, IVIG, plasma exchange, and drugs such as cyclophosphamide or rituximab) following prompt treatment of the tumor may stabilize or slow the progression of the neurologic symptoms [7]. MRI reveals T2 hyperintensity in the limbic structures. Limbic encephalitis can be autoimmune or paraneoplastic [22,23,24,25,26,27,28,29,30]. Limbic encephalitis is rare, and there have not been any randomized-controlled immunotherapy clinical trials [7,23]. T2-weighted fluid-attenuated inversion recovery hyperintensities may be associated with demyelination or inflammation. Early treatment with corticosteroid, intravenous immunoglobulin (IVIG), plasmapheresis, and rituximab may ensure less risk of relapses [7,31,32]. Acute demyelinating encephalomyelitis (ADEM) affects the white matter, whereas lupus cerebritis is associated with cerebral atrophy, small subcortical hyperintensity, and infarct.

NORSE/FIRES or other epilepsy syndromes are diagnosed following exclusion of the aforementioned encephalitis/encephalopathy. We review NORSE and related acronyms and discuss the management and prognosis of these status epilepsy syndromes.

About 10–40% of young patients with SE will develop refractory SE or RSE [33]. RSE is SE that persists in spite of treatment with two or more appropriately dosed parenterally administered medications for at least 60 min, with at least one benzodiazepine [6,34,35,36,37]. Super refractory SE (SRSE) is RSE that continues twenty-four or more hours after anesthesia is induced or recurs after anesthesia is withdrawn [38,39]. The cause is identified within 72 h in most of these patients with RSE. However, RSE may occur in otherwise healthy individuals with no definite etiology in a minority of these cases (i.e., NORSE/FIRES).

Lyon in 1961 described an RSE after a febrile illness with an extremely poor prognosis in previously healthy patients [40]. Many different acronyms have since been described. They include febrile infection or febrile illness-related epilepsy syndrome (FIRES) [41], new-onset refractory status epilepticus (NORSE) [39,42], syndrome of idiopathic hemiconvulsions, hemiplegia, and epilepsy (IHHE) [39,42], devastating epileptic encephalopathy in school-aged children (DESC) [41], and acute encephalitis with refractory and repetitive partial seizures (AERRPS) [43]. FIRES, AERRPS, or DESC are severe and immune-mediated encephalopathies developing in previously healthy pediatric patients [44]. AERRPS and DESC are now obsolete. The lack of unified terminology leads to miscommunication, diagnostic ambiguity, and therapeutic challenges.

A panel group of experts established consensus in defining FIRES and NORSE in 2018 [39]. NORSE is a clinical diagnostic entity and not a specific disease in a patient without any relevant neurologic disorder and an identifiable structural, metabolic, or toxic cause [45]. NORSE includes patients with viral infection or autoimmune causes, or ‘cryptogenic NORSE’ is NORSE without a currently detectable cause but with a suspected one. FIRES is a new-onset RSE associated with a prodromal febrile illness between 24 h and two weeks before RSE onset [39,44,45,46,47]. FIRES is now considered a subcategory of NORSE with fever or NORSEF. Table 1 compares NORSE with FIRES (or NORSEF). Literature reviews a higher mortality in NORSE and a higher male preponderance in FIRES [3,48,49,50]. The two syndromes are otherwise indistinguishable [3].

NORSE and FIRES are both associated with very high mortality and morbidity [55,59]. Therefore, they must be recognized early and treated promptly [4,39,55].

This review provides an update on hypotheses, etiology, pathophysiology, clinical features, diagnosis, laboratory evaluation, treatment, and perspectives for NORSE/FIRES.

## 2. Methods

Using PubMed Clinical Queries, filter “Therapy”, Scope “Broad”, and keywords of “NORSE”, “FIRES”, evidence for the efficacy, management, and prognosis from recent meta-analyses and randomized clinical trials in the NORSE and FIRES in children is reviewed up to May 2025. The knowledge gaps in differentiating NORSE versus FIRES in epidemiology, pathophysiology, clinical features, diagnosis, evaluation, and management are summarized in the following respective headings to illustrate that the two conditions are similar, if not identical, and cannot be clearly differentiated from each other.

## 3. Epidemiology

Worldwide, NORSE has a crude annual incidence of 0.7/100,000 and a 36% mortality rate [52]. Its incidence is unknown among pediatric patients. FIRES affects 1 in 1,000,000 young children [44]. FIRES has been reported in adults [39,45]. It has a male predominance but no known genetic predisposition [55,68]. Seizures in NORSE/FIRES are difficult to treat or recalcitrant.

## 4. Current Hypotheses About Pathogenesis of NORSE and FIRES

The exact pathogenesis of NORSE/FIRES remains unknown. An infectious etiologic agent is rarely identified in FIRES [47]. The current hypotheses suggest a post-infectious, cytokine-mediated inflammatory disorder. Intrathecal overproduction of several chemokines is found in patients with NORSE compared to the cerebrospinal fluid (CSF) of those with other central nervous system inflammatory disorders [69,70]. Certain proinflammatory cytokines and chemokines, namely blood CCL2, CXCL8, and MIP-1α, and CSF IL-1ß, were upregulated, but T cell-associated cytokines were found to be downregulated in children with NORSE [71,72]. Intrathecal inflammation is more prominent in CSF than in serum. This suggests that inflammation in the central nervous system is mediated by humoral immunity.

Furthermore, a reduction in CSF oligoclonal bands and clinical improvement following immunomodulatory treatment have been described in individuals with NORSE and FIRES [73,74,75]. Gene analysis of FIRES patients found an association of a tandem repeat (VNTR) polymorphism of *SCN2A, IL1RN,* and *IL1RN* genes, suggesting multiple genetic factors in FIRES [76]. Lastly, the exclusion of gene mutations associated with fever-sensitive epilepsies, such as the *SCN1A* gene coding for the DNA polymerase subunit gamma-1 (*POLG-1*) and the protocadherin 19 (*PCDH19*) gene, may aid the diagnosis of FIRES [55,67].

## 5. Clinical Features of NORSE and FIRES

Although NORSE has an uncertain etiology, a preceding respiratory illness is often present without any apparent history of fever. On the other hand, FIRES begins with a non-specific febrile infection-like illness in previously healthy children [47]. Seizures usually begin about 24 h to 2 weeks later. Recurrent infrequent seizures occur in the acute phase, typically focal motor ± alteration of consciousness, bilateral generalized or facial/peribuccal myoclonia, and escalating to RSE. In the acute phase, fever may not be present. Profound encephalopathy is present between seizures. A skin rash, cardiac arrhythmia, and impaired liver function may be present in approximately 50% of patients with FIRES [45,77]. The acute phase lasts for several days to a few months [47]. The chronic phase then follows without a seizure-free, or “silent period”. The seizure burden diminishes, and consciousness of the patient gradually improves, but intractable epilepsy, cognitive impairment, and neuropsychological deficits result [77,78]. The mortality rate was reported to be 11.7% in a study of 77 children with FIRES, and 93% of survivors were left with refractory epilepsy. Twelve children (18%) were cognitively intact. Eleven (16%) children had borderline cognition, 10 (14%) had a mild degree of intellectual disability (ID), 16 (24%) children had moderate ID, 8 (12%) children had severe ID, and eleven (16%) children were incapacitated in a vegetative state [45]. In another retrospective series of 29 children reports, 87% had residual ± treatment-resistant epilepsy. Twenty-six percent had a learning disability and mild-to-moderate ID, and 48% had severe ID or a vegetative state [77].

## 6. Pediatrics Versus Adults

NORSE and FIRES present with distinct age-associated differences in genetics, clinical presentation, pathogenesis, and outcomes. Pediatric FIRES typically follow febrile illness within 24 h to two weeks, with a more abrupt onset of seizures and higher seizure burden compared to adults. Children often exhibit prodromal symptoms such as fatigue and gastrointestinal disturbances, whereas adults may present with more subtle cognitive or mood changes [79]. Genetic predisposition in pediatric cases is increasingly linked to immune dysregulation, with studies identifying variants in genes related to innate immunity and cytokine regulation, such as IL1RN and TLR4, although no single pathogenic variant has been universally implicated [80]. Pathogenesis in both NORSE and FIRES involves a hyperinflammatory response, but pediatric patients show elevated levels of proinflammatory cytokines like IL-6 and TNF-α, suggesting a more robust immune activation [80]. Mechanistically, this may reflect age-related differences in blood–brain barrier permeability and neuroimmune interactions. Outcomes also diverge significantly: children with FIRES often suffer long-term cognitive and behavioral impairments, with lower rates of seizure freedom and higher dependence on polytherapy, while adults may retain better functional recovery despite persistent epilepsy [81]. These differences underscore the need for age-specific diagnostic and therapeutic approaches, including early immunomodulatory interventions in pediatric cases. The NORSE/FIRES Family Registry has been instrumental in delineating these distinctions, highlighting the importance of tailored clinical management across age groups [81].

NORSE/FIRES is rare but seems to be more common in children than adults. More research is needed for definite differentiation of the syndrome between pediatrics and adults.

## 7. Clinical Evaluation and Diagnosis of NORSE and FIRES

The diagnosis of NORSE and FIRES is clinical and by exclusion in previously healthy children with ± a preceding febrile illness who develop SRSE, respectively. The initial work-up aims to identify etiologies of refractory SE, including brain infection, trauma, and toxic exposure. In selected cases, autoimmune, genetic, and paraneoplastic causes need to be identified. The diagnosis may often be delayed due to the lack of specific laboratory or imaging markers.

### 7.1. Magnetic Resonance Imaging (MRI)

**Figure 1:**
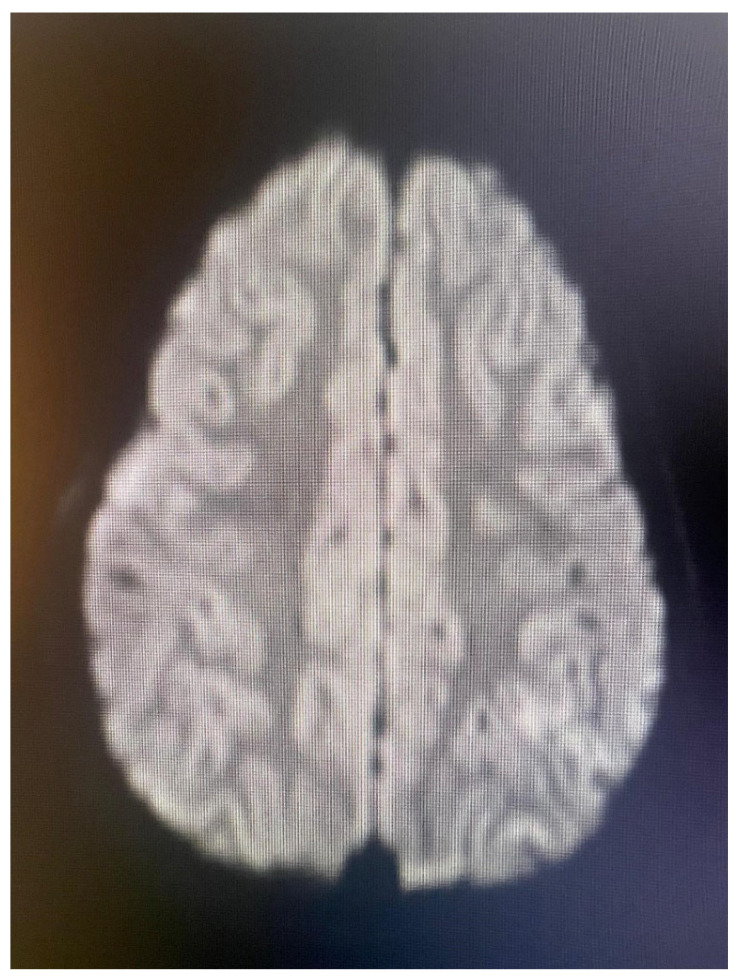
A 6-year-old boy with FIRES (febrile illness refractory epilepsy syndrome), precedent infections include Rhinovirus and Enterovirus. MRI showed foci of gyriform cortical diffusion restriction in the left. parietal lobe and left temporal lobe, with associated relative hypo-enhancement of these cortices, and to a lesser extent at the left hippocampus and right lateral frontoparietal cortex. There is no associated hyper-enhancement or increased FLAIR signal. The MRI findings suggest a diffuse process, but most prominent in the same areas of the EEG.

MRI of the brain may be normal during the acute phase of NORSE/FIRES [45]. Signal abnormalities can be seen in the gray matter of the cerebral cortex, thalamus, basal ganglia, temporal lobes, and hippocampus [82]. Enhancement of the leptomeninges or ependyma can be present [45,73,83]. Three to four weeks after seizure onset, brain MRI may show diffuse atrophy of the cerebella, cerebrum, hippocampal sclerosis, and subcortical temporo-occipital infarcts. These MRI abnormalities may correlate with neurologic outcome (Figure 1) [6,45,77,83,84,85].

### 7.2. Cerebrospinal Fluid and Serum Findings

CSF findings may be normal but may show mild pleocytosis (less than 10 white blood cells) and mild elevation of protein, but usually absent oligoclonal bands in both conditions. There is usually no evidence for metabolic, infectious, autoimmune, or genetic etiologies in the serum or CSF. Proinflammatory cytokines and chemokines have been found in the CSF and serum of children with NORSE/FIRES, suggesting an underlying process of inflammation, but proinflammatory chemokines and cytokines are not routinely clinically performed because they are not specifically diagnostic for NORSE/FIRES [67,70].

### 7.3. Electroencephalogram (EEG) Findings

**Figure 2:**
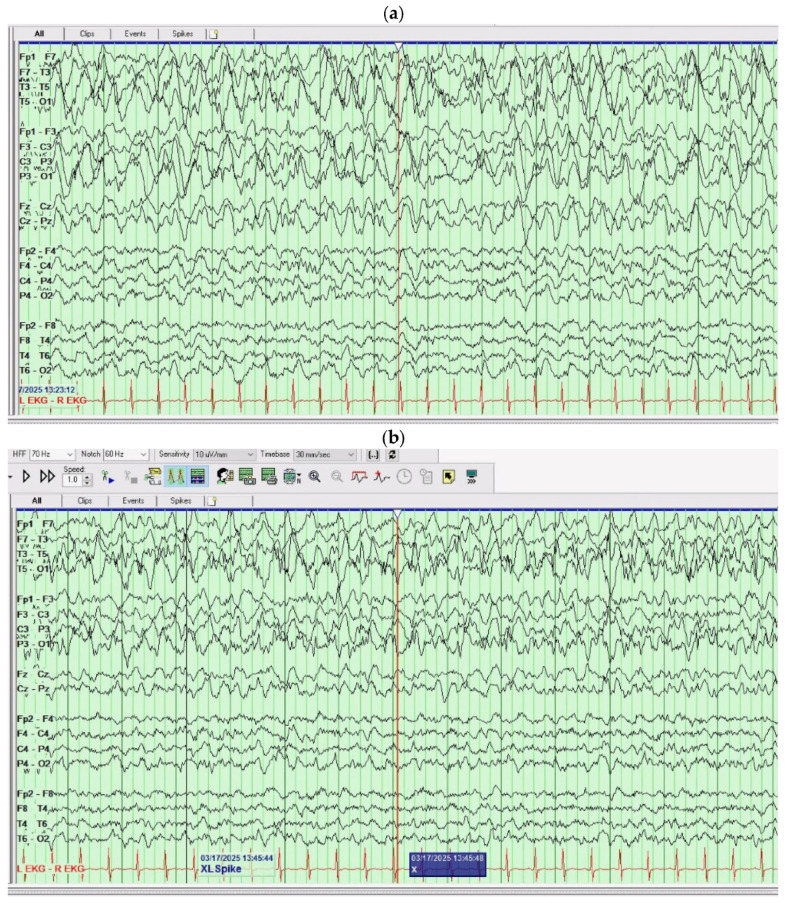
A 6-year-old boy with FIRES (febrile illness refractory epilepsy syndrome), precedent infections include Rhinovirus and Enterovirus. Electrographic (EEG) activity was localized mainly in the left hemisphere. (**a**) EEG on day 1 after admission shows initially frequent runs of left posterior quadrant-posterior temporal/parietal and occipital spike/poly-spike wave up to 9 s, with electrodecrement up to 5 s, either only left hemisphere or diffuse. (**b**) The abnormal 19 h video EEG long-term monitoring remained very active with runs of epileptiform discharges from the left posterior quadrant and then 1 Hz LPD’s left posterior temporal region.

Continuous EEG monitoring is important. Nonspecific findings of multifocal epileptiform discharges and generalized slowing are typically shown, especially in frontal/temporal regions. EEG in NORSE may show focal or generalized periodic and multifocal discharges (Figure 2) [41,45,82]. Seizures are associated with focal fast beta activity that gradually becomes SE with rhythmic spike and wave complexes or beta-delta activity [56]. Farias-Moeller et al. analyzed video EEG recordings in FIRES (n = 14). Three typical EEG signatures during the acute phase are described [56]: (1) Low EEG epileptiform burdens in the initial twelve hours, (2) Stereotypical Extreme Delta Brush (EDB), and (3) EEG seizures with “sparks” (i.e., prolonged low amplitude focal fast activity) followed by rhythmic gradual complexes. These seizures may move from one side to the other side of the cerebral hemisphere. EDB was previously described as a typical EEG pattern in NMDA (Anti-N-methyl-D-aspartate receptor) encephalitis, which is an autoimmune encephalitis with immunoglobulin G antibodies against the NR1 (i.e., NMDA receptor subunit 1 in the CNS [86].

## 8. Management of NORSE and FIRES

There are no good current guidelines to base the clinical management of NORSE/FIRES on [3]. It is very important to consider NORSE/FIRES early in the differential diagnosis of super-refractory status epilepticus (SRSE). The initial treatment of NORSE and FIRES is similar to that for SRSE [3]. Anti-epileptic drug (AED) treatments include barbiturates, burst-suppression coma, cannabinoids, immunotherapy, hypothermia, ketogenic diet, vagal nerve stimulation (VNS), and electroconvulsive therapy (ECT).

Treatment includes ASM, intravenous immunoglobulin, intravenous corticosteroids, burst-suppression coma, and ketogenic diet. Affected individuals often need deep sedation with anesthetic drugs [34,44]. The ketogenic diet may be effective in some cases [4,6,41,45,51,87,88]. VNS is useful in seizure control after recovery from SE [4]. IVIG treatment is not particularly effective for FIRES [89]. Barbiturates are effective in treating SE [90]. Most patients have significant refractory epilepsy and cognitive disability. The treatment of catastrophic encephalitis depends on the underlying etiology, and antiviral medications, anticonvulsive drugs, and corticosteroids may be needed. Severe encephalitis is typically managed in the PICU and with the use of high-dose or pulsed steroids. Pulse IV methylprednisolone therapy is the intermittent administration of supraphysiological quantities of the drug to enhance its therapeutic effects while reducing its toxic effects. A pulse regimen ranges from 500 to 1000 mg IV daily for 2–4 consecutive days. Typically, high-dose (30 mg/kg) pulsed methyl prednisolone followed by a tapering course of steroids is used as an essential part of treatment in these conditions.

### 8.1. Anti-Seizure Medications

Administration of intravenous benzodiazepines such as midazolam and lorazepam is followed by non-benzodiazepine ASM (including phenobarbital, phenytoin, valproic acid, levetiracetam, and lacosamide). In FIRES, SE typically persists regardless of the use of various combinations of conventional ASM. The induction of coma with barbiturates, propofol, midazolam, and inhalational agents is disappointingly non-efficacious, with seizure recurrence following weaning of these agents from the burst suppression coma (BSC) [91]. BSC is the standard approach in SRSE [92,93]. However, seizure activity recurs in the majority of the NORSE/FIRES patients following cessation of BSC [92].

### 8.2. Barbiturates

In FIRES, SE is not responsive to intravenous benzodiazepines and standard first-line anti-epileptics. Second-line treatment involves barbiturate-induced coma [94].

Lowenstein et al. report in a 1988 series that all patients (n = 14) had complete cessation of seizures with barbiturate-induced coma. Six of them died for unrelated reasons [95]. Another series reports that up to half of pediatric patients with FIRES experienced significant side effects from coma induced by barbiturates, and prolonged use of barbiturates was associated with worse clinical outcomes [68]. Barbiturates reduce cerebral blood flow and cerebral edema but lead to hippocampal ischemia [68]. One retrospective study showed that a worse outcome in pediatric FIRES was associated with the duration of barbiturate coma [44]. Nevertheless, very high doses of phenobarbital are efficacious and have fewer side effects than other anesthetic agents [96].

### 8.3. Midazolam

Midazolam (a short-acting benzodiazepine) infusion in anesthetic doses acts by binding to the Gamma-Aminobutyric Acid (GABA) A receptor. Rapid tolerance and breakthrough seizures occurred in 47–57% of patients, typically occurring within hours to days of continuous infusion [38,97,98]. Midazolam has strong respiratory and cardiac depressant effects and is associated with risks of hepatic and renal impairment [38]. Midazolam is as effective as thiopental in treating RSE but with fewer adverse events and is associated with a better long-term neurological outcome [96].

### 8.4. Propofol

Propofol is a short-acting drug that acts via modulation of the GABA (A) receptor. Continuous IV infusion of propofol induces burst suppression coma. The side effects of propofol, including hypotension or cardiocirculatory depression, are less frequent and severe than those of midazolam and the barbiturates [38]. Its prolonged use in critically ill patients and children is associated with the risk of the rare but potentially lethal propofol-related infusion syndrome [PRIS], characterized by hemodynamic instability, bradyarrhythmias, and progressive metabolic acidosis. These complications are often refractory to aggressive treatments. PRIS can develop with low rates of infusion of less than 4 mg/kg per hour. Other late-occurring adverse effects include heart failure, arrhythmia, and electrocardiographic changes [99]. Propofol should not be used for more than 48 h. The drug may induce uncontrolled movements that could be misdiagnosed as myoclonic seizures [100]. The risk for developing PRIS increases when propofol is used with a ketogenic diet.

### 8.5. Ketamine

Ketamine is an alternative intravenous infusion anesthetic drug for SRSE. Ketamine is an N-methyl-D-aspartate (NMDA) receptor channel blocker with possible preferential effects on the NMDA receptors located on GABA neurons. Resolution of RSE in 14 of 19 episodes was shown in 13 pediatric patients with RSE in a report, avoiding endotracheal intubation in five patients in whom ketamine was used in lieu of conventional anesthetics [101]. However, an inconclusive response was reported in other case series where ketamine was used for the pharmacological treatment of FIRES during the acute phase [75,102]. The major theoretical advantage of ketamine infusion is that it has potential neuroprotective action without any cardiac depressant effect [38].

### 8.6. Ketogenic Diet

The ketogenic diet (KD) is the most promising mode of treatment in NORSE and FIRES [103,104,105]. The therapeutic use of a high-fat, low-carbohydrate, low-protein diet KD has been studied in recalcitrant epilepsy for many years since 2003 [106]. Classically, a ketogenic diet is a 4:1 ratio of fat to combined carbohydrate and protein. Only enteral ketogenic diet administration has been studied in children with FIRES [87]. These pediatric patients recovered consciousness within 48 h after seizure cessation. Motor functions recovered within the ensuing weeks. KD continued for 6–24 months. All patients developed infrequent seizures and chronic epilepsy. Another retrospective review on 10 children between 2 and 16 years of age with SRSE, including NORSE and FIRES, reports that nine of the patients had resolution of the SRSE after commencing the classic ketogenic diet [63]. Another case series of nine children with SRSE found that anesthetic infusions were weaned off within 1 week of a ketogenic diet [83]. Singh et al. reported SRSE was successfully abolished in two pediatric patients with FIRES by a KD in the acute SE phase and subsequently with mild cognitive impairment [105]. Currently, early introduction of the ketogenic diet within the initial few days of SRSE and continuation of the diet into the chronic phase are recommended. Rare metabolic conditions such as pyruvate carboxylase and beta oxidation deficiencies must be excluded before starting KD. Lack of dieticians with expertise in the ketogenic diet, parenteral formulation, protocol standardization, difficulties with enteral feeding, and interfering conditions such as co-administration of steroids and phenobarbital are technical challenges.

### 8.7. Immune Therapy

First-line drugs include intravenous steroids, intravenous immunoglobulins, and plasma exchange. Second-line drugs include anakinra, rituximab, and tocilizumab.

#### 8.7.1. Steroids, IVIG, and Plasma Exchange

Recent findings of upregulation of proinflammatory cytokines in CSF and autoantibodies to voltage-gated potassium channels suggest an inflammatory-mediated pathophysiologic mechanism for NORSE/FIRES [107]. Overall, there are over 100 cryptogenic NORSE and 200 cryptogenic FIRES cases reported in the literature that received immune pharmacological treatments during the acute or chronic phase. These therapies include steroids, intravenous immune globulin (IVIG), and plasmapheresis, suggesting a partial or unclear response [55]. Data are only available from small uncontrolled series and case reports with report bias and with no randomized controlled trial. Hence, there is no current consensus regarding the use and efficacy of any of these immune therapies in NORSE and FIRES. Nevertheless, these reports have shown better outcomes with immunotherapies in adults [44]. Typically, high-dose (30 mg/kg) pulsed methylprednisolone followed by a tapering course of steroids is used as an essential part of the treatment of SE [108].

#### 8.7.2. Immunomodulating Agents

*Anakinra*. Anakinra is a recombinant human interleukin-1 (IL-1) receptor antagonist used to treat several disorders of autoimmunity. IL-1 is a cytokine involved in inflammatory cell recruitment. Currently, there has been only one case report on treatment with anakinra in a 32-month-old female patient with refractory status epilepticus due to FIRES [64,65]. The girl had increased cytokine levels in the CSF prior to treatment. Treatment with anakinra was useful, well-tolerated, and without any side effects [64,65,103].

*Rituximab*. Rituximab is a humanized B-cell depleting antibody that has been used for SRSE and chronic refractory epilepsy [3,58]. A case report shows an 11-year-old female patient with NORSE and associated anti-GAD 65 encephalitis who had an unsatisfactory response to standard anti-epileptic drugs and standard immunotherapies [109]. She was treated with intrathecal dexamethasone and rituximab and improved with reduced seizure burden [109]. Side effects of rituximab include infections, tiredness, body aches, nausea, and infusion-related reactions [110,111].

*Tocilizumab.* Tocilizumab is an anti-interleukin-6 humanized monoclonal antibody that acts against the IL-6 receptor. In a series of patients with NORSE (n = 7) with refractory seizures, SE was terminated after tocilizumab in six patients [66,112]. Another series showed that tocilizumab was associated with measurable early EEG reduction in epilepsy activities in NORSE [113]. Earlier administration of the medication after SE onset correlated with a shorter duration of SE [103].

### 8.8. Cannabinoids

Epidiolex is a cannabidiol approved for treating recalcitrant epilepsies in children with Lennox–Gastaut and Dravet syndromes. The medication is potentially an add-on drug for NORSE and FIRES [114]. Gofshteyn et al. reported Epidiolex use in seven patients with recalcitrant FIRES. Seizures improved in six patients [115]. Potential side effects associated with the use of Epidiolex include lethargy, loss of appetite, mood changes, diarrhea, transaminitis, and elevation of clobazam levels [114]. Use of high-dose cannabidiol (CBD) in the NORSE/FIRES adult case was reported to be efficacious, with significant improvement in activities of daily living [116].

### 8.9. Magnesium Sulfate

Magnesium blocks the NMDA receptor channel; hence, there may be similar activity as with ketamine. Infusion of magnesium sulfate has been used in treating seizures [38]. One study reports the use of continuous magnesium sulfate infusion in two children with FIRES, and only one partially responded [117]. Further studies are necessary before the use of magnesium sulfate infusion can be recommended for NORSE/FIRES.

### 8.10. Vagal Nerve Stimulation (VNS)

Vagal nerve stimulation (VNS) has been used for pharmaco-resistant epilepsy. The favorable impact of VNS on RSE and generalized seizures has been reported [38]. Zeiler et al. conducted a systematic review (n = 17 studies) on the use of VNS for RSE [118]. VNS demonstrated better efficacy on seizure reduction in generalized RSE (76%) than in focal RSE (25%). The effect of VNS on RSE (n = 16 children) was reviewed and demonstrated that the frequency of all seizure types decreased in twenty percent of patients, and generalized convulsive seizures decreased in nearly seventy percent of patients at one year after VNS implantation. The role of VNS therapy for RSE/SRS in pediatric patients (n = 7) is similar [61]. Prompt VNS use is suggested for patients with resistant RSE/SRSE to provide rapid responses and reduce pharmacological load. In NORSE/FIRES, VNS provides neuromodulation during the acute phase if standard treatments are ineffective. This approach may leverage VNS’s potential anti-inflammatory effects and neuromodulation and enhance patient-specific treatments.

### 8.11. Electroconvulsive Therapy (ECT)

Electroconvulsive therapy (ECT) has been found to be useful in patients with RSE in case reports and case series [119]. ECT terminated RSE in a pediatric patient with FIRES, who failed treatments with multiple ASMs, high-dose barbiturates, ketamine, and a ketogenic diet. Rescue use of ECT was reported to be useful in a pregnant woman diagnosed with NORSE [120]. Further studies are needed to evaluate the usefulness of ECT in children with RSE, NORSE, and FIRES.

### 8.12. Moderate Therapeutic Hypothermia

Therapeutic hypothermia (TH) reduces proinflammatory cytokines in the brain.

It stabilizes immune activation, protects the blood–brain barrier, reduces brain edema and seizures, and prevents ongoing damage in the brain. Fast sustained seizure control with moderate therapeutic hypothermia (33 °C) in two patients with FIRES was described [121]. TH is generally not recommended for NORSE due to a lack of evidence.

### 8.13. Miscellaneous

Other approaches, including lidocaine, transcranial magnetic stimulation and epilepsy surgery, are not established and therefore not elaborated here [122,123].

## 9. Future Directions

Current treatments for NORSE/FIRES are based on expert opinions and extrapolated from case reports and small series in RSE. The efficacy of various benzodiazepines is confirmed. All second-line treatments (e.g., fosphenytoin and levetiracetam) are equally effective. Non-anti-seizure medications aiming at underlying pathophysiology should be considered in SE. Immunosuppressant therapies are widely used for NORSE/FIRES. EEG monitoring in ICU setting with automated analysis of the EEG could aid assess the control of electrographic seizures [46]. Early KD may optimize seizure control and improve cognitive outcome in patients with NORSE/FIRES.

NORSE and its subtype FIRES are rare but devastating neurological emergencies characterized by prolonged seizures unresponsive to standard treatments. Recent advances in understanding their immunological underpinnings have prompted clinical trials exploring targeted immunotherapies. A major international clinical trial, funded by the Patient-Centered Outcomes Research Institute (PCORI), is currently underway across 33 centers [124]. This study compares the efficacy of two interleukin inhibitors—anakinra (IL-1 receptor antagonist) and tocilizumab (IL-6 receptor antagonist)—in improving outcomes for patients with NORSE and FIRES. The trial aims to determine which agent more effectively reduces seizure burden and improves neurological recovery, potentially transforming the standard of care for these conditions. This trial is grounded in emerging consensus recommendations published by an international panel of experts, which emphasize early immunotherapy initiation, multidisciplinary care, and standardized diagnostic protocols. The study also incorporates patient-centered outcomes, including quality of life and long-term cognitive function, to better understand the broader impact of these therapies. The trial’s design reflects a shift toward precision medicine in epilepsy care, recognizing the inflammatory cascade as a key therapeutic target. The trial could pave the way for regulatory approval of immunomodulatory agents in NORSE/FIRES and inform future trials exploring adjunctive therapies such as ketogenic diets or neurostimulation.

The conventional approach for pediatric intractable epilepsy has been the use of ASM with a similar mechanism of action in combinations. However, antiepileptics mainly block neurotransmitter receptors or affect neuronal ion channels. Therefore, the use of multiple ASMs is seldom efficacious for seizure reduction in NORSE/FIRES. New approaches to the treatment of NORSE/FIRES come in a timely manner when the condition is more clearly delineated, and the approaches to diagnosis are better formulated. Nevertheless, large-scale and multicenter controlled studies will provide evidence for better treatment guidelines for these rare conditions, with multicenter registries and the use of biobank data to improve patient management. Furthermore, NORSE/FIRES are not distinct syndromes and should be considered one disease entity. Since 2012, the NORSE Institute has developed a family-based registry [39]. The goals of this registry are to provide support to families by sharing patient and family experiences and encouraging research. Also, there is an ongoing collaboration between the NORSE Institute and the European Reference Network for rare and complex epilepsies to develop the NORSE/FIRES registry [39,48,55,125].

FIRES is now considered a subset of NORSE. The diagnosis is clinical and based on New Onset (NO) and Status Epilepticus (SE) in a previously healthy child, with SE that persists despite ≥two appropriately selected and dosed parenteral anti-epileptic drugs. Therefore, we propose a new acronym, NOSES, to replace the NORSE/FIRES acronym. Following the NOSES diagnosis, treatment is as follows:

1. Anti-seizure medications ± anesthesia-induced coma ± antimicrobials;
2. Supportive treatment with airway, breathing, and cerebral support;
3. Ketogenic diet ± vagus nerve stimulation;
4. EEG monitoring;
5. Iatrogenic damage avoidance;
6. Immunotherapy with anakinra, tocilizumab, etc.

## 10. Conclusions

The major knowledge gaps include gaps in understanding etiology, pathophysiology, diagnosis, and specific treatment for the NORSE/FIRES, especially in pediatrics.

We support the recommendations from the NORSE Institute and the European Reference Network for rare and complex epilepsies that it is necessary to use standard terminology and diagnostic approaches for NORSE/FIRES. The definition and diagnostic approach of NORSE/FIRES should be updated timely. The exact etiology of NORSE/FIRES is still not known. There remains an urgent need to develop guidelines based on controlled clinical trials using patient registry data. A NOSES diagnosis and EASI approach are proposed.

## Figures and Tables

**Table 1:** NORSE versus FIRES [48,49,50,51].

	NORSE	FIRES
Incidence	0.7/100,000 per annum [52]	1 in 1,000,000/yr (children) [47]
Risks	Young age, female, previously healthy, cerebrospinal fluid pleocytosis, antecedent febrile illness, extraordinarily prolonged status epilepticus, no apparent underlying cause despite extensive investigations, the catastrophic outcome, and temporal lobe plus leptomeningeal abnormality on MRI of the brain [52,53].	Males more commonly affected than females [45]
Etiology	Proinflammatory molecules in the brain following upper respiratory viral infection [5,52,54].	A preceding upper respiratory tract or gastroenteritis one day to two weeks before onset.
Pathophysiology	- Cryptogenic in 52% of cases [5] - Nonparaneoplastic autoimmune (anti-NMDA receptor antibody) - Paraneoplastic causes	Uncertain [55]
Diagnosis	CSF: Inflammatory pleocytosis without an identifiable organism [5,53]. EEG: - Lateralized or focal, generalized periodic discharges, and multifocal discharges [41,45]. - Seizures are brief and infrequent at the onset, associated with focal fast beta activity with a gradual evolution to SE characterized by rhythmic spike and wave complexes or beta-delta activity [56]. - In 1/3 of the anti-NMDA receptor encephalitis cases, EEG monitoring may show beta bursts overriding delta waves [57]. Neuroimaging: - Hyperintensity on T2/FLAIR sequences within the limbic and neocortical structures, often bilaterally. - Other brain regions like the claustrum, basal ganglia/thalami, and peri-insular cortex show abnormal T2/FLAIR hyperintensity [41,58].	By exclusion: - The acute phase consists of highly recurrent focal seizures, rapidly evolving into refractory status epilepticus. The chronic phase consists of drug-resistant epilepsy with cognitive impairment. CSF: Pleocytosis EEG: - FIRES is a focal process with focal onset seizures. In a 2011 study of 77 FIRES patients, 58 had focal seizures. Of the 58, 50 had secondarily generalizing seizures (seizures that evolve from focal to generalized) [45,50]. On a 10–20 scalp electrode EEG, the ictal activity commonly begins temporally and spreads hemispherically and/or bilaterally [59]. Interictally, patients may have slowing that may be considered an encephalopathic pattern [60]. - FIRES patients (n = 12): diffuse delta-theta background slowing interictally in all 12 cases [58].
Treatment	- Benzodiazepines, ASM [53] - Anesthetics for induced coma (with a median of 5 antiseizure drugs during treatment, yet 77% of cases culminate with administration of continuous anesthetics [5] - 40% of status epilepticus cases will be refractory to the first and second-line treatments [5] - Anesthetic use is associated with poorer outcomes and increased mortality [5]. - Vagus nerve stimulation efficacious in some [61,62].	Benzodiazepines, barbiturates, and ketogenic diet [45,51,63]. Anakinra or Tocilizumab [64,65,66]
Prognosis	50% of survivors develop chronic cognitive or functional disability and epilepsy [5].	Intellectual disability, behavioral problems, and ongoing seizures [45,67].
Mortality	36% [52]	12% [45]

FIRES was named in 2010 by Andreas van Baalen and colleagues [47,48]. Previous names include AERRPS (acute encephalitis with refractory, repetitive partial seizures), DESC (Devastating Epilepsy in School-aged Children), and NORSE (New-Onset Refractory Status Epilepticus) [38,54].

## Data Availability

No new data were created or analyzed in this study.

## Abbreviations

ADEM	Acute Disseminated Encephalomyelitis.
AERRPS	Acute Encephalitis with Refractory, Repetitive Partial Seizures.
DESC	Devastating Epilepsy in School-aged Children.
FIRES	Febrile Infection-Related Epilepsy Syndrome.
IHHES	Idiopathic Hemiconvulsion-Hemiplegia and Epilepsy Syndrome.
NORSE	New-Onset Refractory Status Epilepsy.
RSE	Refractory Status Epilepticus.
SE	Status Epilepticus.
VNS	Vagal Nerve Stimulation.
